# Effects of transsectoral long-term neurorehabilitation

**DOI:** 10.1186/s42466-023-00302-3

**Published:** 2024-02-08

**Authors:** Mareike Schrader, Annette Sterr, Tobias Strank, Stephan Bamborschke, Christian Dohle

**Affiliations:** 1 P.A.N. Zentrum für Post-Akute Neurorehabilitation, Wildkanzelweg 28, 13465 Berlin, Germany; 2 School of Psychology, University of Surrey, Guildford, GU2 7XH UK; 3 Center for Stroke Research Berlin, Charité – University Medicine Berlin, 10117 Berlin, Germany

**Keywords:** Neurorehabilitation, Long-term rehabilitation, Stroke, Acquired brain injury, Chronic phase, Transsectoral

## Abstract

**Background:**

Acquired brain injuries are among the most common causes of disability in adulthood. An intensive rehabilitation phase is crucial for recovery. However, there is a lack of concepts to further expand the therapeutic success after the standard rehabilitation period. Hereafter, the characteristics of a transsectoral, multiprofessional long-term neurorehabilitation concept and its effects on outcome at different ICF levels are described.

**Methods:**

The P.A.N. Center for Post-Acute Neurorehabilitation combines living with 24/7 support of pedagogical staff with on-site outpatient therapy and medical care. A secondary data analysis was conducted on the records of all patients with completeted P.A.N. treatment between 01.01.2015 and 09.04.2022. Outcome parameters included demographic characteristics, diagnostics, Barthel Index (BI), the German scale „Hilfebedarf von Menschen mit Behinderung für den Lebensbereich Wohnen “ (HMBW), the Canadian Occupational Performance Measure (COPM) and the destination after discharge. For BI and discharge destination, potential determinants of therapy success are evaluated.

**Results:**

168 patients were enrolled in the analyses. Significant improvements were observed in the BI (*p* < .001), with median values increasing from 55 to 80 points. The HMBW showed a significant decrease in the need for assistance in *everyday living* (*p* < .001), *individual basic care* (*p* < .001), *shaping social relationship* (*p* = .003) and *communication* (*p* < .001). Significant improvements were reported in the COPM total score for performance (*p* < .001) and satisfaction (*p* < .001). 72% of the patients were able to move in a community living arrangement with moderate need for support. Main predictive factor for discharge destination was the initial cognitive deficit. The comparison of the third-person scales BI and HMBW with the self-reported COPM showed that individually formulated patient goals are only insufficiently reflected in these global scales.

**Discussion:**

The data show that a highly coordinated, trans-sectoral 24/7 approach of goal-oriented practice as pursued at P.A.N. is feasible and effective. We assume that the success of the intervention is due to the high intensity of therapies delivered over a long time and its interlink with real world practice. For a comprehensive analysis of rehabilitation success, it is necessary to record and evaluate individual patient goals, as these are not always reflected in the commonly used global scales.

**Supplementary Information:**

The online version contains supplementary material available at 10.1186/s42466-023-00302-3.

## Background

Neurological conditions are among the leading causes for disability in adulthood [1–3]. Neurorehabilitation can reduce the permanent consequences of acquired brain injury. The sustainability of the rehabilitation success depends on an early, consistent and intensive therapy [4]. In Germany a specific comprehensive neurorehabilitation system was developed, the so called “neurological phase model”, providing different rehabilitation settings for patients with different levels of disability [5, 6]. But even within this system, inpatient rehabilitation is only funded for a limited period of time and mainly based on continuous improvements of basic activities of daily living (ADL) functions, e.g. as measured with the Barthel Index (BI). For those patients with the slowest recovery trajectory this time can be too short to fully exploit their rehabilitation potential [7, 8]. This can lead to the (false) conclusion that these patients are beyond further improvement. Moreover, training in the rather artificial setting of a rehabilitation unit can only provide an incomplete preparation for life within the community post-discharge. It is for these reasons, that Page and co-workers called for new approaches to facilitate further recovery over 20 years ago [8].

After discharge from inpatient rehabilitation into the community setting, different goals can be formulated. The primary goal is to maintain the functional progress and to prevent further decline which is frequently observed [9]. For patients on a slower recovery path, further improvements should be promoted. In principle this is possible even in an outpatient setting. However, in reality treatment densities drop progressively after discharge [10, 11]. A survey in Germany showed that only about one quarter of patients receive extensive and almost one quarter a low amount of physical and occupational therapy. Over 50% did not receive any outpatient treatment at all [11]. Besides, the outpatient system (community setting) is fragmented and poorly coordinated [12]. Most of the time, the actors involved like general practitioners, neurologists, therapists, nursing care, and integration support are working independently from each other. In this phase, it is often up to the patients and their relatives to demand and coordinate appropriate therapies. These factors jeopardise the maintenance or improvement of the previously achieved therapy success.

**Fig. 1:**
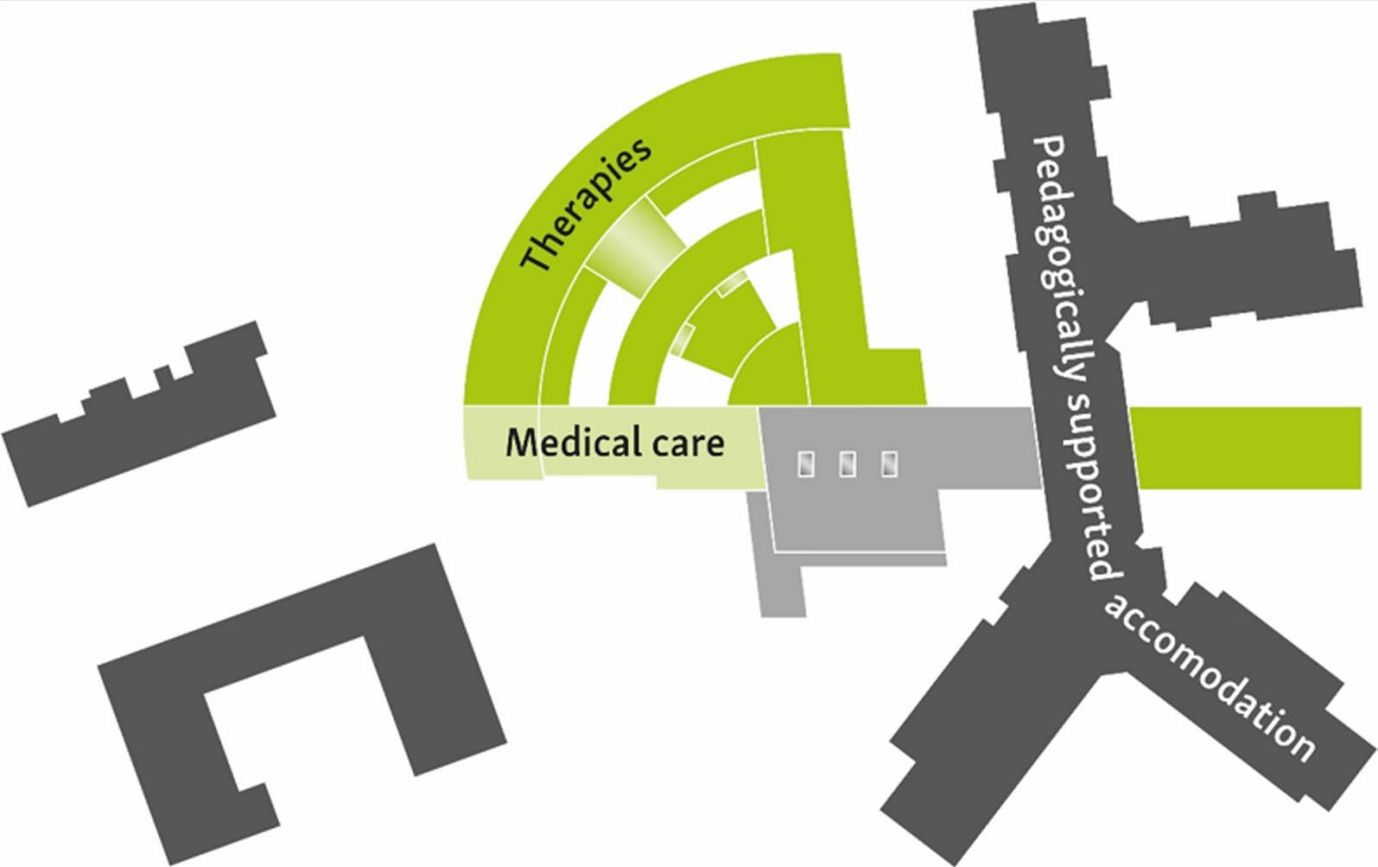
Schematic display of the components of the P.A.N. centre

In the following, a new rehabilitation concept pursued at the P.A.N. Centre for Post-Acute Neurorehabilitation (P.A.N.) is described. The concept tries to resolve the aforementioned issues. Key to this concept is the combined provision of outpatient medical care, therapy and pedagogic supervision in a 24/7 program under a single roof (see Fig. 1). The overarching goal of this setting is the optimal preparation of patients for independent living. The present study therefore examined various outcome parameters at different ICF [13] levels to determine the benefits of this concept. Assessment-based markers were evaluated and contrasted with the patients’ perspective.

## Methods

### Concept

The P.A.N. accommodates up to 66 patients of working age, living in groups of up to 14 individuals. They live together in a flashare-type setting where each patient has their own room but kitchen, living room etc. are shared. Importantly, these ‘flatshare groups ‘are supervised by pedagogically trained staff (e.g. learning disability nurse, rehabilitation support worker). Their role is to teach patients all matters of daily living through guided practice and enabling support. Moreover each patient is assigned to one member of pedagogic staff as personal key worker to support this person throughout his or her entire stay. In addition, the P.A.N. complex houses a rehabilitation centre, where therapies such as occupational therapy, physiotherapy, speech and language therapy and neuropsychology are provided. Neurological supervision and day-to-day medical needs are met by three neurologists. Additional care needs are covered by external GPs and other medical services as required. Regular multi-professional team meetings take place to track progress and review goals. Particular emphasis is thereby placed on the close coordination of pedagogic and therapeutic goal content.

The funding model underpinning the pedagogical and therapeutic components of the concept are provided by different strands of the German health care- and social services systems (mainly integration support and health insurance schemes). According to the specific insurance scheme, there are some differences in therapy intensity and content, but these issues are not covered in the following analysis.

### Study population

A secondary data analysis was conducted on the records of all patients whose treatment at P.A.N. was completed between 01.01.2015 and 09.04.2022. During the observation period, there has been a continuous update in therapy content, but no systematic change of the total rehabilitation concept. Written informed consent for data mining was obtained from patients or their legal representative if appropriate. The data access procedure was approved by the local data protection officer.

### Outcome parameters

Outcome parameters comprised demographic characteristics, diagnostics, and the presence of speech disorder and cognitive disorder, respectively (as assessed by ICD coding at admission). For assessments, the BI [14, 15] and Early Barthel Index (ERBI) [16] indices, the German scale „Hilfebedarf von Menschen mit Behinderung für den Lebensbereich Wohnen “ (HMBW) [17], and the Canadian Occupational Performance Measure (COPM) [18] were used. These measure were chosen to reflect the areas of function, activity and participation of the International Classification of Functioning, Disability and Health (ICF) [13]. A further variable, discharge destination, was constructed for the living arrangements patients moved to after the treatment.

The BI [14, 15] is a measure for the ICF domain *activity*. It describes ADL and serves to systematically assess independence and need for care. Ten items are categorized if patients can perform the task independently, with some assistance, or without help, or not at all, scoring 0, 5, 10 or 15 points respectively. The total score ranges from 0 (complete dependence) to 100 (full independence) points. Additionally the ERBI [16] was obtained. This scale lists seven deficits that are scored with negative values if present. The maximum score is -325 points. Due to the patient sample, the present study only includes the two items on *severe communication deficit* and *severe orientation disorder*. BI and ERBI were collected quarterly by pedagogic staff. For the present analysis, the data of the first and last quarter were analysed.

The HMBW [17] assesses assistance needs of people with disabilities, and captures both the *activity* and the *participation* domain of the ICF model. It consists of 34 activities divided into seven domains. In the present study, 25 items of the domains *everyday living, individual basic care, shaping social relationships, participation in cultural and social life, communication and orientation* were included. For each item the need for assistance is rated on a 4-point scale (1: no assistance required, 2: information/assistance needed, 3: deputy execution/accompaniment required, 4: comprehensive assistance). The HMBW was assessed by pedagogic staff. For this analysis, we used the data after admission and before discharge.

The COPM [18] captures the patients’ subjective performance and satisfaction of self-set goals over time. It consists of five steps: 1. Definition of problems the patient seeks improvements for (goals), 2. rating their importance, 3. selecting the five most important problems for scoring, 4. scoring performance and satisfaction on a 10-point scale (1 indicating poor performance/low satisfaction, respectively, while 10 indicates very good performance/very high satisfaction), and 5. reassessment by the patient after some time. The COPM assessment was conducted in the first quarter of the rehabilitation by occupational therapists. The collection of the COPM was systematically introduced in 2017, thus these data were only available for a subset of the sample.

The variable ‘discharge destination’ describes if patients succeeded in moving into a community living arrangement or were still dependent on intensive support such as in a nursing home. Germany offers a wide range of housing options with various levels of support. These options were grouped into the categories *community living with moderate need for support, community living with enhanced need for support, intensive personal care required *and *other* (see Additional file 1). For the binary logistic regression analysis described below, the discharge destination grouping was restricted to two categories: *living arrangement with 24-h professional support* and *living arrangement without 24-h professional support* (see Additional file 1).

### Statistical analysis

Statistical analyses were conducted using SPSS Statistics 28.0, graphics were created with Excel or Python. Descriptive statistics comprised means (M) and standard deviation (SD), medians (MD) and interquartile range (IQR), and frequencies (N; %) for continuous (normally distributed) and categorical variables, respectively. Within-group changes over time were compared by the Wilcoxon Rank-Sum Test (WRST; paired samples).

In order to analyse the influence of other variables on the BI, a multilevel model (MLM) was calculated, comprising a macro-level factor (patients) and a micro-level factor (repeated measure). A fixed effects model was used to assess the influence of factors; a random effect model to examine individual differences between patients. Factors included were time (t1; t2), age at admission, gender, diagnosis, time since onset (TSO), length of stay (LOS) and the interaction of time*LOS. To analyse predictors for the discharge destination, a binary logistic regression was calculated. To analyse the influence of predictors on the need for 24-h support after discharge (yes/no), a generalized linear model was used to estimate the Odds Ratios of the predictors to fall in the category of 24-h support. The main effects included were: gender, age at admission, TSO, LOS, diagnosis, BI at admission, presence of speech disorder and cognitive disorder. For all statistical analysis, a significance level of 5% (two tailed) was set.

## Results

### Study population

During the study period, 214 potential participants were identified. Consent was not available for 40 patients, for a further six patients assessments were incomplete. This left a sample of 168 patients for BI and HMBW analyses, and 74 for the COPM. Table 1 shows their demographic data at admission. In both groups, participants were predominantly male and around 48 years of age. More than 60% suffered from stroke. On average, patients were admitted 8 months after the event and stayed for 18 months in the centre. During that time, they received a median of 7.7 h of therapy per week.

**Table 1:** Demographic data at admission

		Full sample (N = 168)	COPM Subgroup (N = 74)
Age (years)	Md (IQR)	47.5 (36;54)	48.5 (36;55)
Gender	N (%)	♀ 56 (33.3) ♂ 112 (66.7)	♀ 29 (39.2) ♂ 45 (60.8)
*Diagnosis*	N (%)		
Traumatic brain injury Stroke Other		31 (18.5) 107 (63.7) 30 (17.9)	14 (18.9) 47 (63.5) 13 (17.6)
Time since onset (month)	Md (IQR)	8 (6;12)	7 (5;11)
*Transferred from*	N (%)		
Rehabilitation clinic Residential unit (nursing care) Home Other		138 (82.1) 12 (7.1) 11 (6.5) 7 (4.2)	66 (89.2) 2 (2.7) 4 (5.4) 2 (2.7)
Barthel Index admission	Md (IQR)	55 (45;80)	55 (45;75)
Cognitive disorder (yes)	N (%)	106 (63.1)	45 (60.8)
Speech disorder (yes)	N (%)	109 (64.9)	43 (58.1)
Length of stay (month)	Md (IQR)	18 (12;23)	19 (15;23)
*Therapies (minutes per week)*	Md (IQR)		
Physiotherapy Occupational therapy Speech therapy Neuropsychology		145 (119;181) 127 (103;162) 106 (40;154) 85 (58;100)	148 (120;181) 131 (106;161) 100 (27;151) 84 (67;102)

### Effect on function and activity (Barthel Index)

**Fig. 2:**
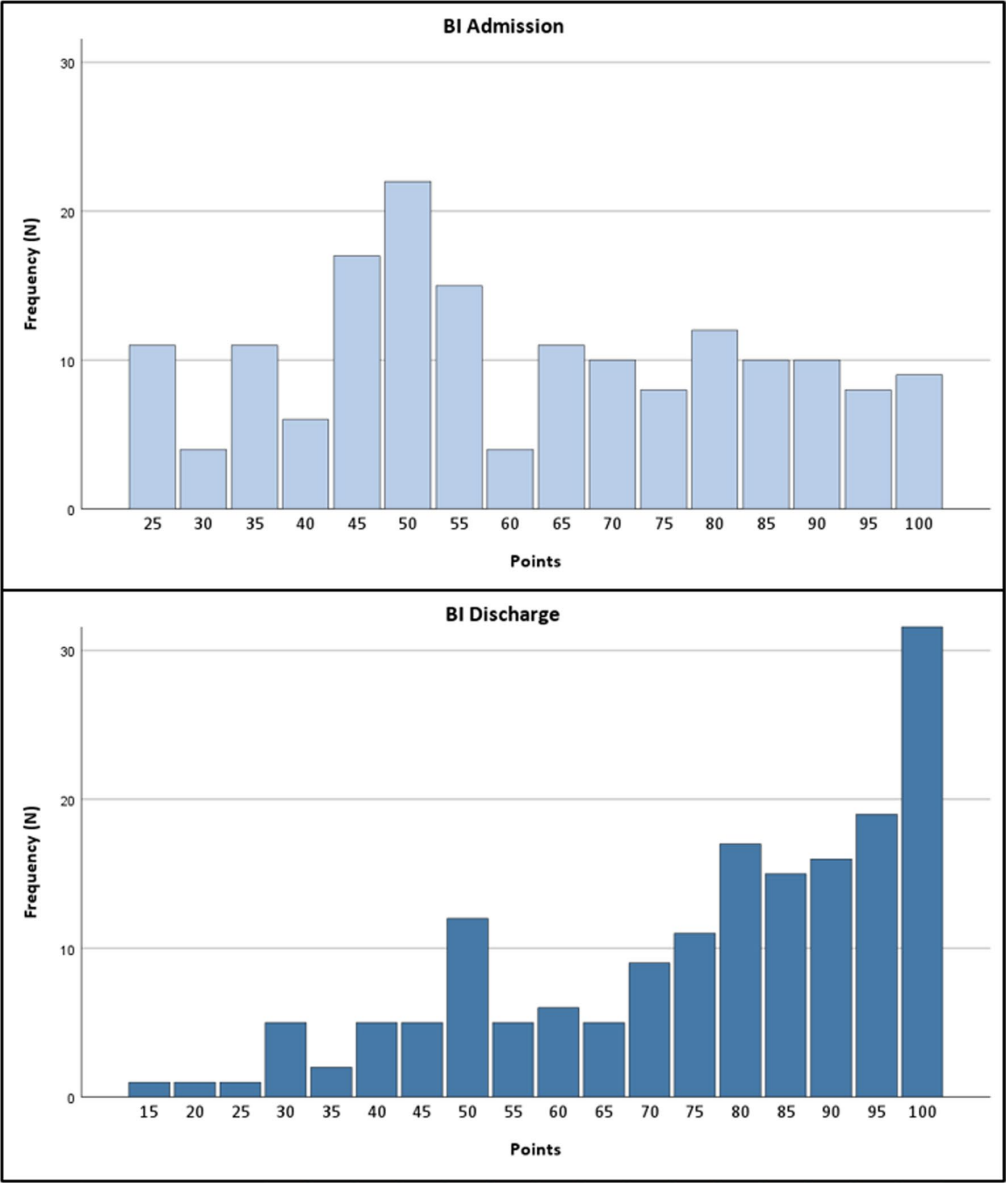
Barthel Index at admission and discharge (histogram, N = 168)

The average time between T1 and T2 for the BI was 16.5 (SD 8.3) months. At admission, the BI was almost normally distributed with a median value of 55 points. At discharge, a strong shift towards the maximum score is apparent with more than 70% achieving at least 70 points (see Fig. 2). This improvement is highly significant (WRST: z = − 9.180, *p* < 0.001).

At the level of individual BI items, improvements were observed for all ten items (see Additional File 2). Most patients struggled initially with *bathing*, *climbing stairs* and *grooming*. Over 50% got better in the areas of *transfer*, *toilet use* and *grooming*. *Climbing stairs* and *mobility* showed the smallest increases (approximately 20%), although almost half of the patients were able to walk when they moved out. More than 40% of patients with orientation disorders and 30% with communication deficits improved during the stay.

In the MLM cognitive disorder and speech disorders at admission could not significantly improve the model and were therefore not included. In contrast, the 2-way interaction “time*LOS” improved the model (*p* < 0.001) significantly and was therefore included (see Additional file 3). The MLM identified TSO as a significant influencing factor on the clinical development of the BI (*p* < 0.001). However, the influence was relatively small (decrease of 0.58 BI points per month). The effect of LOS was not significant (*p* = 0.43), but in interaction with T2 it showed a slight upward trend. All other modelled effects were not significant.

### Effect on activity and participation (HMBW)

**Fig. 3:**
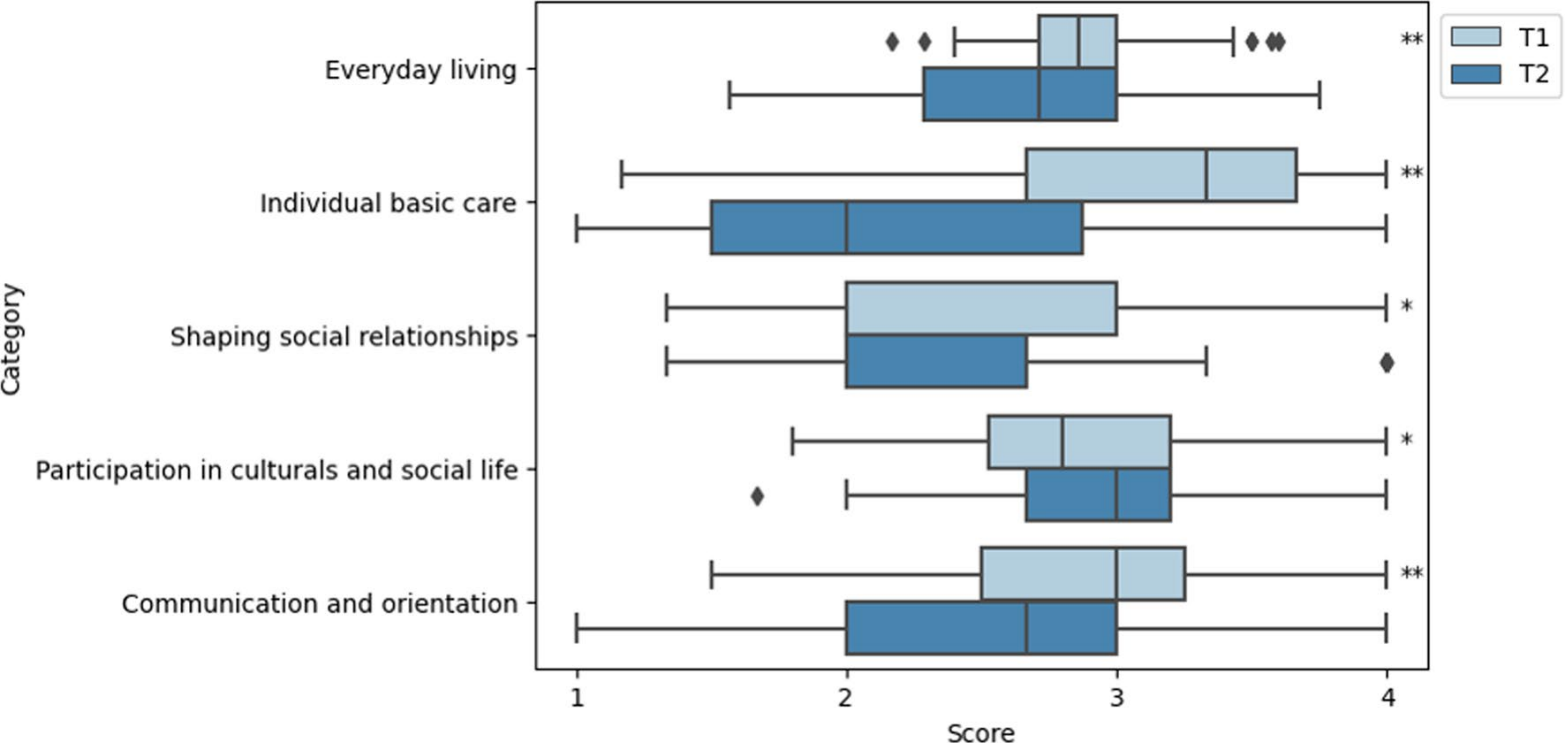
Activity/participation profile (categories of the HMBW) at admission and discharge (median values/IQR of the averaged HMBW, N = 168). Lower values indicate less need for assistance, scoring values as explained in the text. *p < .05; **p < .001 (WRST)

The time between the two surveys averaged 16.3 (SD 8.3) months. Figure 3 shows the need for support at domain level. Four out of five domains show a decrease in the need for assistance (*everyday living p* < 0.001; *individual basic care p* < 0.001; *shaping social relationship p* = 0.003; *communication p* < 0.001). The strongest improvements (22%) were recorded in the domain of *individual basic care.* Within this domain, the items *Toileting/personal hygiene* and *get up/go to bed* are particularly noteworthy: 45% and 38% of the patients who previously required assistance in these items no longer needed or requested help (see Additional file 4). On the other hand, the need for support in the area of *participation in cultural and social life* even increased significantly (*p* = 0.012).

### Patient perspective (COPM)

**Fig. 4:**
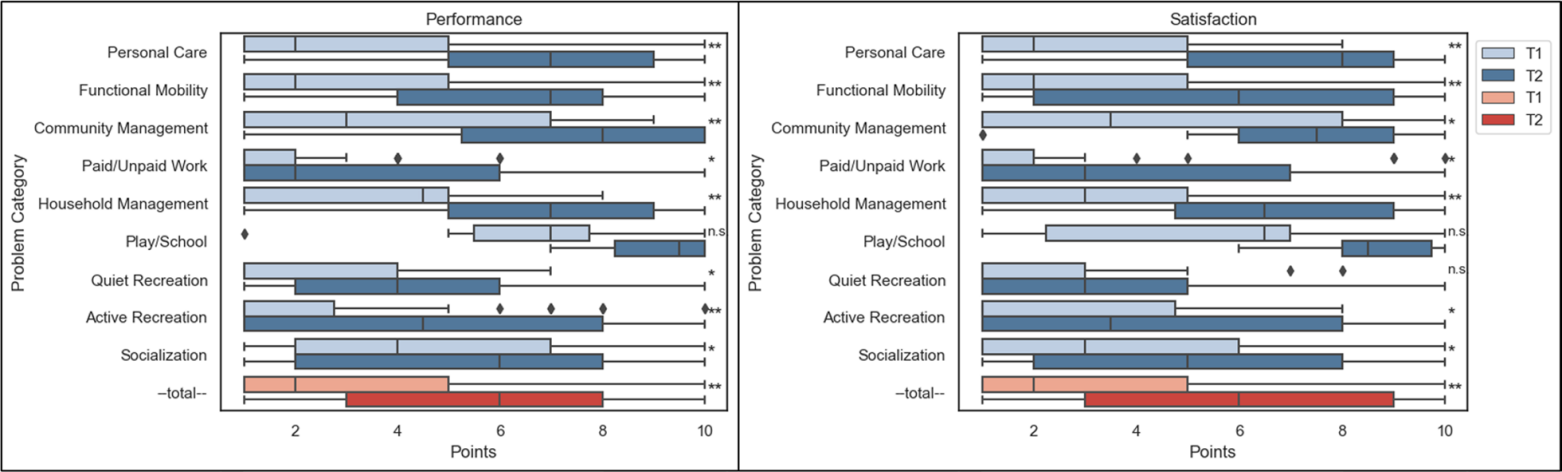
Patient reported performance and satisfaction in the subcategories (blue) and total value (red) of the COPM (Median values/IQR, N = 74). Higher values indicate more improvement/satisfaction. *p < .05; **p < .001 (WRST)

The COPM was introduced about two years into the study period, therefore only 74 cases could be included in this analysis. The average time between the assessment (T1) and reassessment (T2) was 17.5 (SD 10.8) months. At T1, the problems that patients mainly nominated as personal goals were *functional mobility* (26.5%), *personal care* (21.8%) and *household management* (14.5%) (see Additional file 5). In contrast, the domains *work* (9.2%) and *leisure* (< 8.4%) were less frequently mentioned. At T2, significant improvements in self-rated performance were reported in eight out of nine domains and the total score (*p* < 0.001). Satisfaction improved significantly in seven out of nine domains and the total score (*p* < 0.001) (see Fig. 4), implying that patients got better with those problems that they had personally prioritized.

In order to see how many patients with deficits identified through the assessments conducted by the pedagogic staff actually nominated these as their own rehabilitation goals, single items of the BI and HMBW were mapped to the COPM domains (see Additional file 6). This comparison showed a substantial mismatch, with only three out of the nine COPM domains mapping well with the BI and seven out of nine with the HMBW. In all matching domains, the number of patients naming these domains as their personal goals was well below the number of patients with objectively assessed deficits (see Table 2). This shows that both assessments were unable to fully capture personal goals.

**Table 2:** COPM distribution of the five most important problems compared to objective assessments (N = 74)

Problem categories COPM	Patients who nominate this as min. 1 of 5 goals N (%)	Patients who have deficits in this area according to the BI N (%)	Patients who have deficits in this area according to the HMBW N (%)
*Self-care*			
Personal care	45 (61)	68 (91.9)	73 (98.6)
Functional mobility	59 (79.7)	67 (90.5)	72 (97.3)
Community management	17 (23)	–	74 (100)
*Productivity*			
Paid/unpaid work	29 (39.2)	–	–
Household management	36 (48.6)	31 (41.9)	74 (100)
Play/school	4 (5.4)		–
*Leisure*			
Quiet recreation	17 (23)	–	74 (100)
Active recreation	27 (36.5)	–	74 (100)
Socialization	27 (36.5)	–	74 (100)

### Discharge destination

**Fig. 5:**
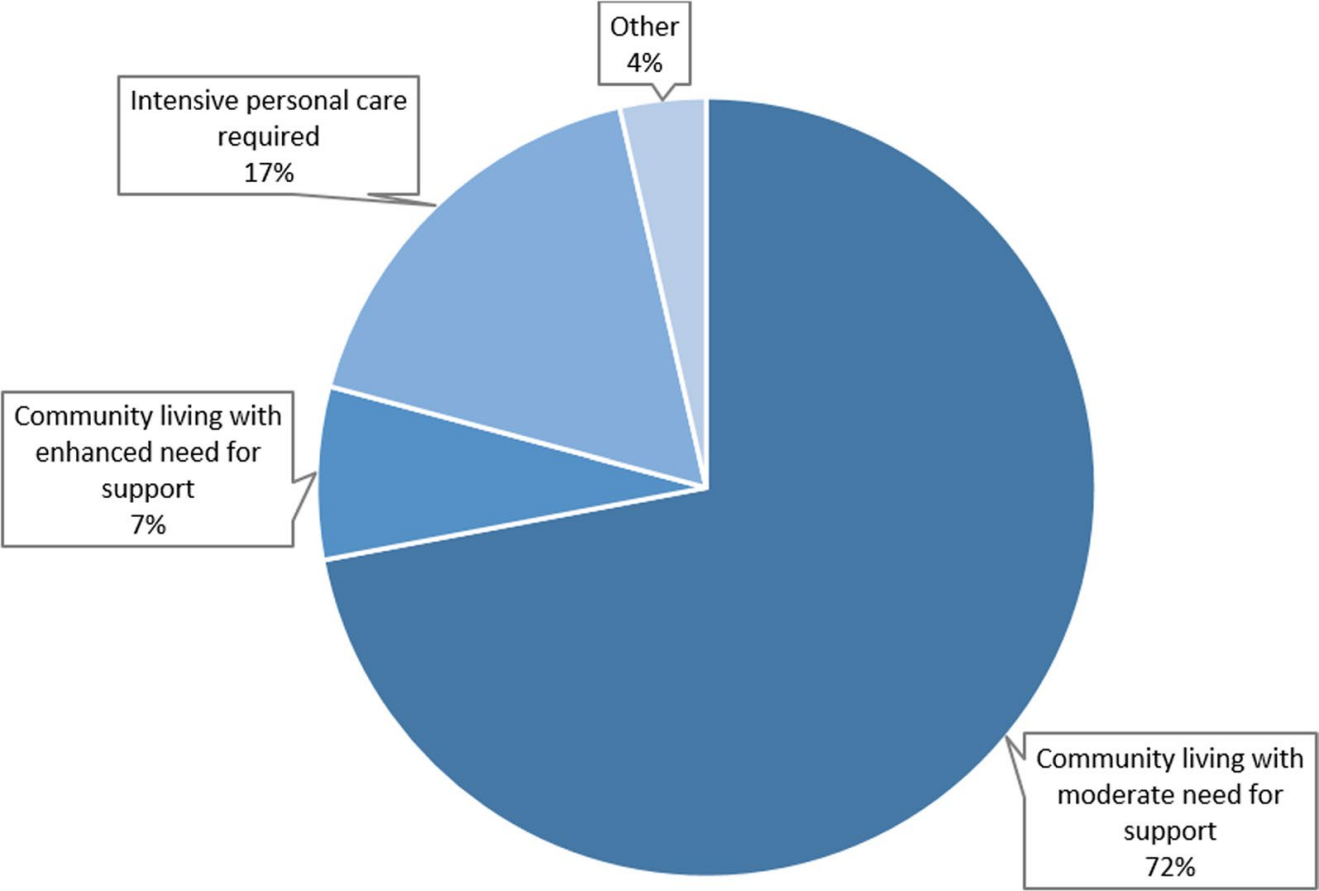
Destination after discharge from the P.A.N. centre (N = 168)

More than 70% of the patients were able to move in a community living arrangement with moderate need for support (see Fig. 5). Only 17% had to move to an inpatient setting where intensive personal care is provided.

The binomial logistic regression performed to identify predictors of discharge into a living arrangement with 24-h support was carried out with 162 cases, since 6 cases could not be assigned to either category (rehabilitation clinic N = 2, discharge destination not known N = 3, death N = 1). The regression model was statistically significant (χ^2^(9) = 42.837, *p* < 0.001) with an adequate effect, as shown by Nagelkerke’s R^2^ = 0.343. Of the eight variables entered into the regression model, two contributed significantly: BI (*p* < 0.001) and cognitive disorders (*p* = 0.002). The risk for discharge into a living arrangement with 24-h-support decreases to 0.947 times per increasing point on the BI at admission, (95%—CI [0.924, 0.971]), corresponding to 0.76 times (0.947^5^) for 5 points increments, i.e. one BI item. The risk of moving into a living arrangement with 24-h care increases 5.045-fold with the presence of cognitive impairment at admission. All model coefficients can be found in Additional file 7.

## Discussion

The data presented above provide strong evidence for measurable and meaningful improvements in patients who had sustained severe chronic deficits despite previous extended in-patient rehabilitation. This suggests that the highly interprofessional coordinated trans-sectoral 24/7 approach of goal-oriented practice pursued at P.A.N. is feasible as well as effective. We observed that the treatment at P.A.N. had clear benefits not only on objectively assessed activity and participation (BI, HMBW), but also regarding self-assessment (COPM). However, these assessments do not fully overlap. The discharge statistics further show that the P.A.N. treatment substantially reduced the risk to need round-the-clock professional support, thus helping many patients into a more independent form of living. The main predictive factor for discharge destination was the initial cognitive (rather than motor) deficit.

### Effects on basic ADL functions

The significant improvements of ADL function appear especially remarkable because it is often assumed that the rehabilitation potential diminishes in the chronic stage. A cohort study by Meyer and co-workers [9], for example, showed that functional and motor outcomes increased within the first six months after stroke (including rehabilitation), but returned to the level measured at two months at the five-year follow-up. Older age and increased severity of disability correlated negatively with a positive recovery rate. Meyer and co-workers concluded that there is a need to improve long-term outcome.

The patients in the present study are younger but similarly impaired and showed excellent ADL gains obtained through a much longer period of post-acute neurorehabilitation. Remarkably, the vast majority of the patients had already received intensive rehabilitation previously. Previous research showed small differences in ADL abilities only for longer time in rehabilitation with higher therapy intensity [19, 20]. The median therapy intensity at P.A.N. averaged 7.7 h per week, thus certainly higher than the usual outpatient frequency in Germany. Moreover, with its interlinked concept of intensive therapy and pedagogically supervised living, additional training takes place in a realistic setting and therefore fosters real-world transfer effects. A good example for this intrinsic link is doing laundry. This activity trains arm function and action planning, increases participation and is scheduled in the weekly therapy plan. Training of meaningful activities outside therapy times prevent sedentary behavior as described in many rehabilitation facilities [21]. Our data clearly show that intensive long-term rehabilitation can still expand skills after “standard” rehabilitation. However, in our MLM analysis, we observed no effect of LOS on the BI, but an interaction effect with even lower BI values at t2. We assume that the non-normal distribution of the BI at t2 with a clear ceiling effect contributes to this statistical effect. Other studies have also shown that patients have unmet rehabilitation potential, highlighting the lack of appropriate services [7, 8, 22, 23].

### Effect beyond basic ADL functions

Given the focus of the P.A.N. concept on activity and participation beyond basic ADL functions, it seems surprising that improvements on the participation scale (HMBW) are less pronounced. The analysis of the patients’ perspective (COPM) gives some insights into this effect. The COPM data show that the individually nominated goals are quite different between patients, and that patients clearly report success in these areas. However, summing up the 25 items of the global HMBW scale does not, perhaps unsurprisingly, capture the individuality of those goals. This is a very important observation since patient-centered goal setting has become a central component of rehabilitation practice [24], yet outcome measures for quality assurance typically rely on broad assessment tools. The limitations arising from the current assessment system is particularly illustrated by our data on household management: at admission, all patients have limitations in this area, but only less than half of them nominate improvements as their personal goal. These patients report high improvements (COPM), but this is not reflected in the objective assessment of the entire group (HMBW). These data highlight the importance of choosing methodologies that restrict measures to the areas featuring as personal goals and hence being areas of focus for treatment [23]. Within the quality control for geriatric rehabilitation of the German health insurance system (“QS-Reha”), a suitable, broadly applicable method has been developed and is presently evaluated [25].

The P.A.N. concept was designed to enable patients to be discharged into a form of living that is as independent as possible. Analysis of the respective data showed that approximately three quarters of the patients achieved this goal. Interestingly, the level of BI at admission is marginal for the outcome on discharge while the presence of neurocognitive dysfunction is a main driver for this outcome. The influence of neurocognitive disorders on the mode of living after stroke was also highlighted by others [26, 27]. In the present study, age does not play a major role. However, we noticed that there was a tendency for the older patients in our sample to prefer a setting where increasing levels of assistance would be available in the future should their health decline in subsequent years.

### Limitations and further outlook

The present study has some limitations. First, the results cannot be contrasted with a control group. It would be conceivable to use data of similarly affected patients and their development for comparison. For the analyses of influencing factors of the BI and the discharge destination we used cognitive disorders at admission as an independent variable. The presence of a cognitive impairment was frequently taken from the discharge report of the previous facility. However, we assume that the abilities have not changed substantially between discharge and P.A.N. admission. Furthermore, we did not include previous (pre-event) living circumstances and marital status in the regression analysis, even though it is known that these factors can influence the discharge destination [27].

Admittedly, the outcomes found in this study cannot unreservedly be generalized to the wider stroke population because P.A.N. patients are selected thorough a strict evaluation process, especially with regards to age and their ability to actively participate in the high intensity program. Besides, not all eligible patients got financing by the different health and social services. For a relevant proportion of the patients, even this funding does not cover the entire costs. Some of the shortfall is currently met by the charitable Fürst Donnersmarck foundation, thus this system cannot readily be replicated elsewhere. One of the funding deficits arises from the extra time required for the continuous multiprofessional coordination of goal content. This coordination effort is not funded by the German outpatient system despite it being essential for rehabilitation success [28]. As shown, communication between the different professions is highly likely to improve outpatient care. The results of this study may further strengthen the need for adequate funding of professional exchange during the long-term treatment of patients with acquired brain injuries.

## Conclusion

The data show that transsectoral long-term rehabilitation with a holistic approach can induce impactful and meaningful improvements even in the chronic phase after acquired brain injury. The success of the intervention is most likely due to both the high treatment intensity and the interlinked provision of goal-specific therapy with real world practice.

### Supplementary Information

**Additional file 1**. Categorisation of discharge destination for graphical representation and regression analysis

**Additional file 2**. Need for support at admission and discharge of the BI and ERBI single items in % (N=168). “Full ability not present” indicates any value below full scoring in the respective category

**Additional file 3**. Regression coefficients and their tests of significance of the MLM (dependent variable BI; details depicted in the text)

**Additional file 4**. Need for support at admission and discharge of the HMBW single items in % (N=168). Support needs ranging from information/assistance to comprehensive assistance (as explained in the text)

**Additional file 5**. Ranking order of the five most important problems as nominated in the COPM (N (%), N=74). Note that not every Patient named five problems, thus the total number of problems is below 5*74=370

**Additional file 6**. Correspondence of BI and HMBW single items to the COPM categories. Mapping not possible for BI: Bladder control, Bowels control; HMBW: develop perspectives on life in the future, Domain communication and orientation

**Additional file 7**. Regression coefficients and their tests of significance for the discharge destination (dependent variable living arrangement with 24-hour support; details depicted in the text)

## Data Availability

The datasets supporting the conclusions of this article are included within the article and its additional files.

## Abbreviations

ADL: Activities of daily living
BI: Barthel Index
CI: Confidence interval
COPM: Canadian Occupational Performance Measure
ERBI: Early Barthel Index
HMBW: Hilfebedarf von Menschen mit Behinderung für den Lebensbereich Wohnen
ICF: International classification of functioning, disability and health
IQR: Interquartile range
LOS: Length of stay
M: Mean
MD: Median
MLM: Multilevel model
P.A.N.: Centre for Post-Acute Neurorehabilitation
SD: Standard deviation
TSO: Time since onset
WRST: Wilcoxon rank-sum test
