# Supplementary figures and images for: Effects of transsectoral long-term neurorehabilitation
Source: Neurol Res Pract. 2024 Feb 8;6:7. doi: 10.1186/s42466-023-00302-3 (PMC10851455; doi:10.1186/s42466-023-00302-3)

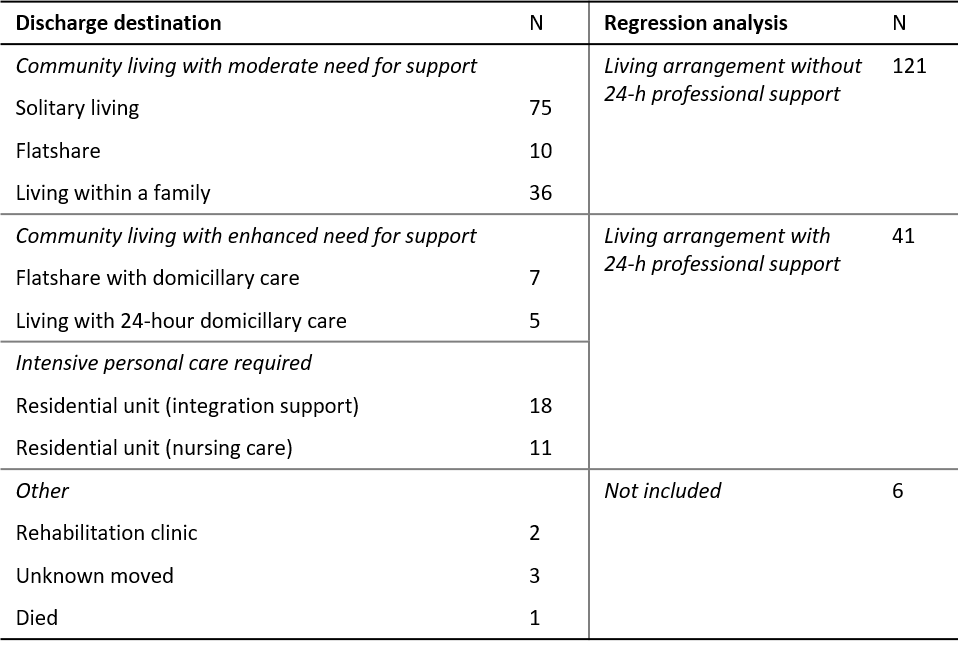

Supplement: Supplementary file 1 — Additional file 1. Categorisation of discharge destination for graphical representation and regression analysis [file 42466_2023_302_MOESM1_ESM.tif]

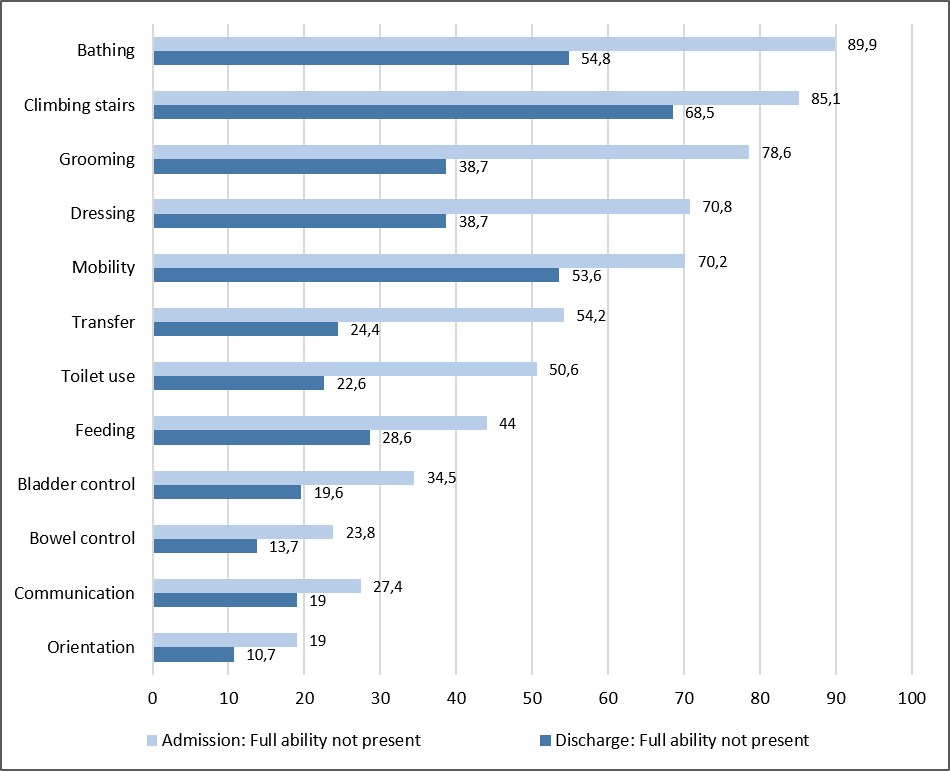

Supplement: Supplementary file 2 — Additional file 2. Need for support at admission and discharge of the BI and ERBI single items in % (N=168). “Full ability not present” indicates any value below full scoring in the respective category [file 42466_2023_302_MOESM2_ESM.tif]

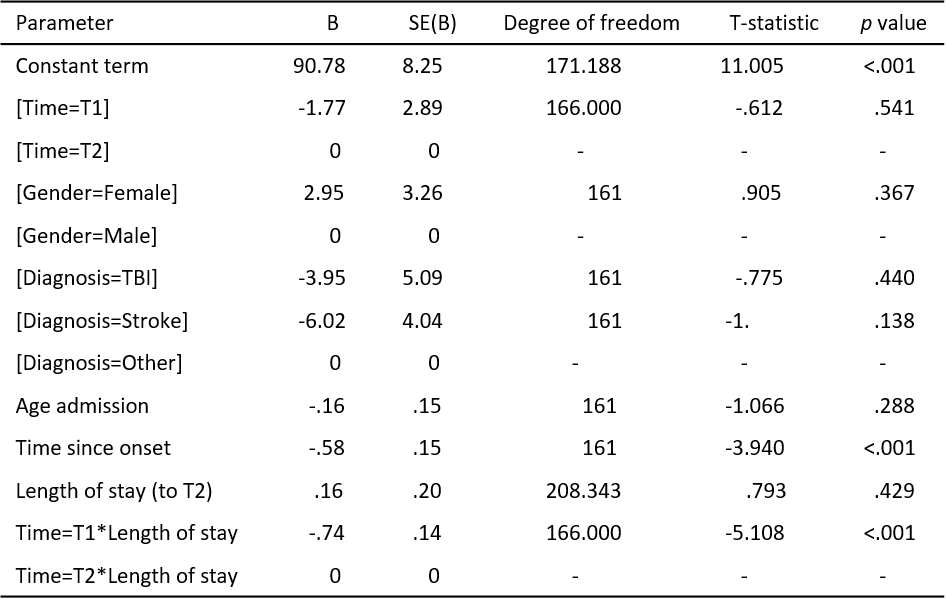

Supplement: Supplementary file 3 — Additional file 3. Regression coefficients and their tests of significance of the MLM (dependent variable BI; details depicted in the text) [file 42466_2023_302_MOESM3_ESM.tif]

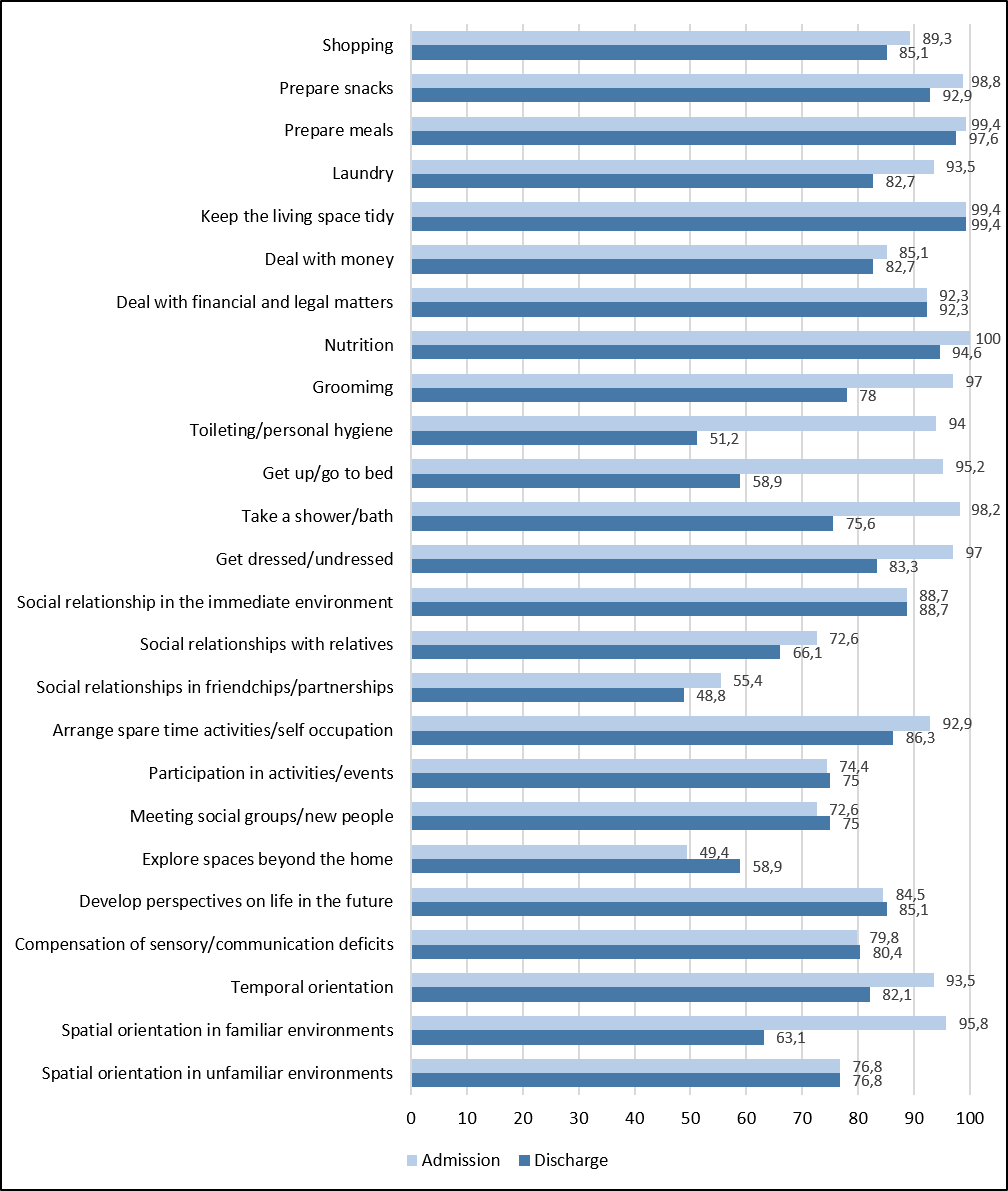

Supplement: Supplementary file 4 — Additional file 4. Need for support at admission and discharge of the HMBW single items in % (N=168). Support needs ranging from information/assistance to comprehensive assistance (as explained in the text) [file 42466_2023_302_MOESM4_ESM.tif]

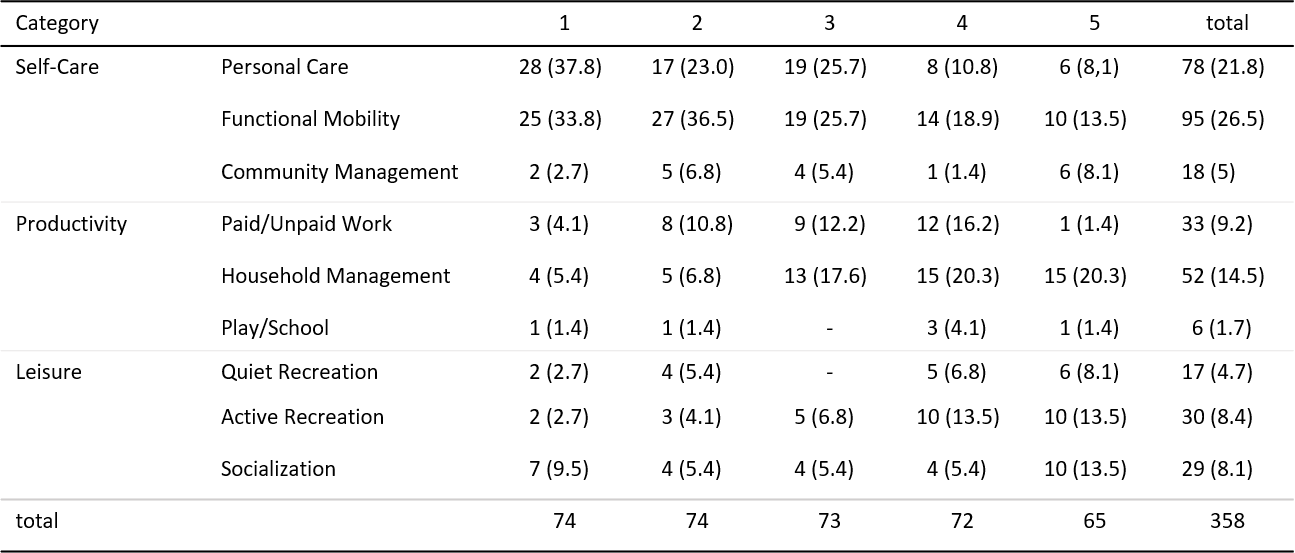

Supplement: Supplementary file 5 — Additional file 5. Ranking order of the five most important problems as nominated in the COPM (N (%), N=74). Note that not every Patient named five problems, thus the total number of problems is below 5*74=370 [file 42466_2023_302_MOESM5_ESM.tif]

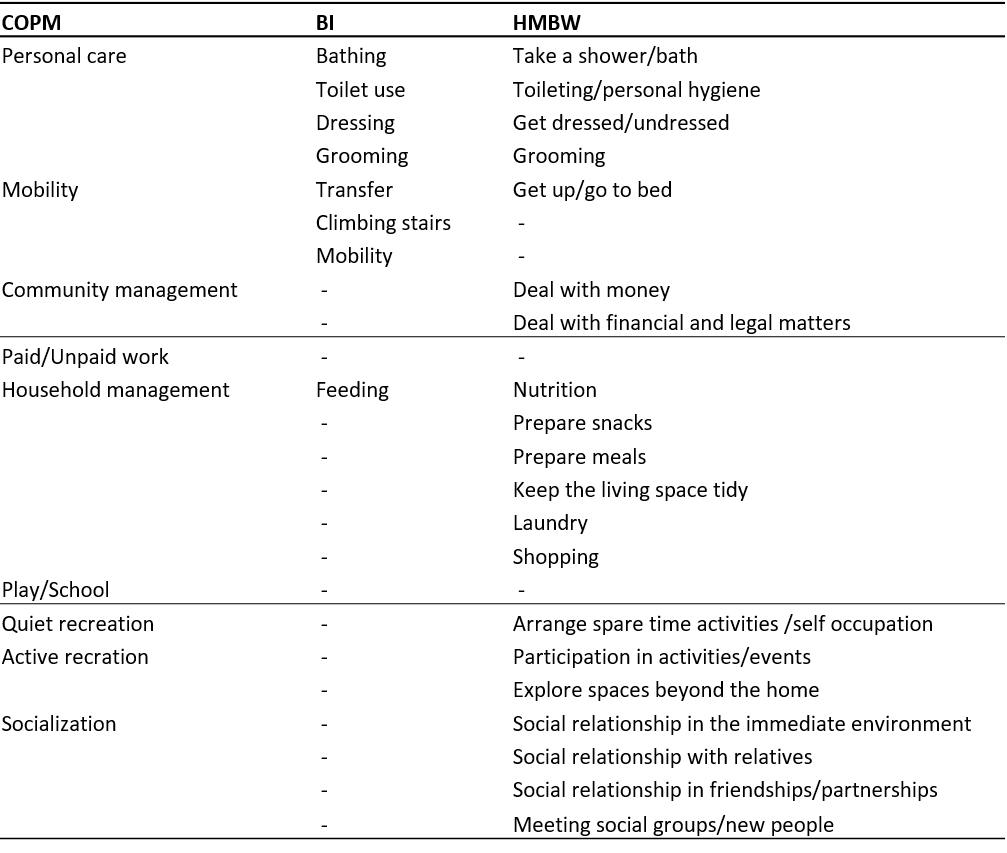

Supplement: Supplementary file 6 — Additional file 6. Correspondence of BI and HMBW single items to the COPM categories. Mapping not possible for BI: Bladder control, Bowels control; HMBW: develop perspectives on life in the future, Domain communication and orientation [file 42466_2023_302_MOESM6_ESM.tif]

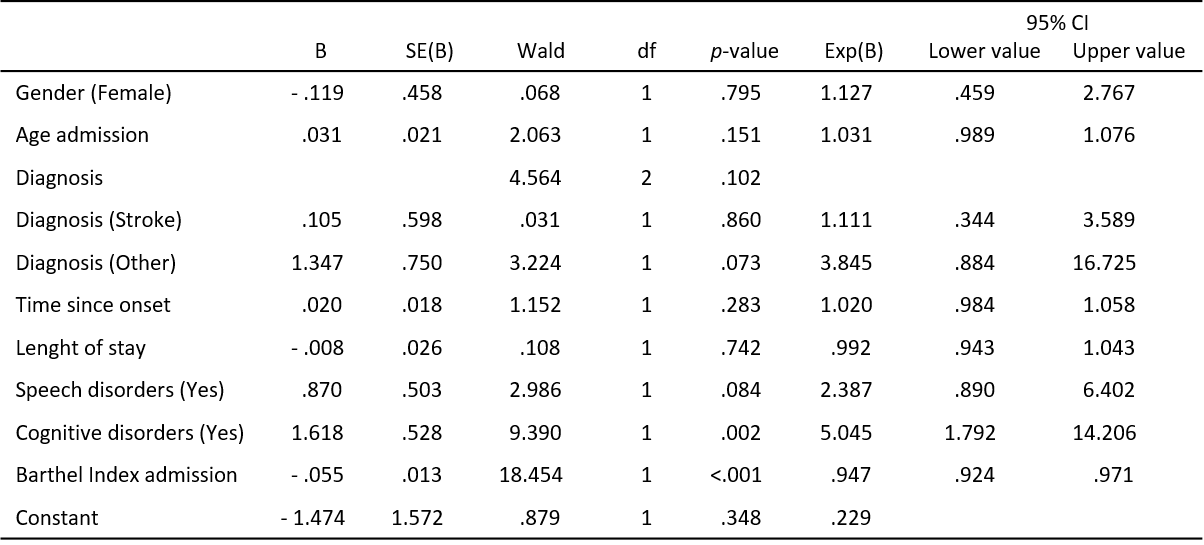

Supplement: Supplementary file 7 — Additional file 7. Regression coefficients and their tests of significance for the discharge destination (dependent variable living arrangement with 24-hour support; details depicted in the text) [file 42466_2023_302_MOESM7_ESM.tif]
